# Factors contributing to cognitive improvement effects of acupuncture in patients with mild cognitive impairment: a pilot randomized controlled trial

**DOI:** 10.1186/s13063-021-05296-4

**Published:** 2021-05-12

**Authors:** Jae-Hong Kim, Myoung-Rae Cho, Jeong-Cheol Shin, Gwang-Cheon Park, Jeong-Soon Lee

**Affiliations:** 1 Department of Acupuncture and Moxibustion Medicine, College of Korean Medicine, DongShin University, Naju City, 58245 Republic of Korea; 2 Department of Nursing, Christian College of Nursing, Gwangju City, 61662 Republic of Korea; 3 Clinical Research Center, DongShin University Gwangju Korean Medicine Hospital, 141, Wolsan-ro, Nam-gu, Gwangju City, 61619 Republic of Korea

**Keywords:** Acupuncture, Mild cognitive impairment, Randomized controlled trial

## Abstract

**Background:**

Mild cognitive impairment (MCI) is generally regarded as the borderline between cognitive changes of aging and very early Alzheimer’s disease (AD). It is important to develop easily available interventions to delay the progression of MCI to AD. We investigated factors contributing to the cognitive improvement effects of acupuncture to obtain data for developing optimized acupuncture treatments for MCI.

**Methods:**

This outcome assessor-blinded, randomized controlled trial included a full analysis for comparing the efficacy of different acupuncture methods. Thirty-two participants with MCI (i.e., fulfilling the Peterson diagnostic criteria for MCI, K-MMSE scores of 20–23, and MoCA-K scale scores of 0–22) were randomly assigned to basic acupuncture (BA; GV20, EX-HN1, GB20, and GV24 for 30 min), acupoint specificity (AS; adding KI3 to BA), needle duration (ND; BA for 20 min), or electroacupuncture (EA; electrical stimulation to BA) groups (*n*=8/group) via 1:1:1:1 allocation and administered acupuncture once daily, three times a week for 8 weeks. The measured outcomes included scores on the Korean version of the Alzheimer’s Disease Assessment Scale-cognitive subscale (ADAS-K-cog), Korean version of the Montreal Cognitive Assessment scale (MoCA-K), Center for Epidemiological Studies-Depression Scale, Korean Activities of Daily Living scale, Korean Instrumental Activities of Daily Living scale, and European Quality of Life Five Dimension Five Level Scale. Outcome measurements were recorded at baseline (week 0), intervention endpoint (week 8), and 12 weeks after intervention completion (week 20).

**Results:**

Twenty-five patients with MCI completed the trial (BA group, 8; AS group, 6; ND group, 5; EA group, 6). MoCA-K scores were significantly increased in the BA group compared with the ND (*p*=0.008, week 8–week 0) and EA groups (*p*=0.003, week 8–week 0; *p*=0.043, week 20–week 0). ADAS-K-cog scores were significantly decreased in the BA group compared with the ND group (*p*=0.019, week 20–week 0).

**Conclusions:**

The BA group showed significant improvement in cognitive function compared to the ND and EA groups. Electrical stimulation and needle duration may contribute to the cognitive improvement effects of acupuncture in patients with MCI.

**Trial registration:**

Clinical Research Information Service; URL:cris.nih.go.kr.; unique identifier: KCT0003430 (registration date: January 16, 2019).

**Supplementary Information:**

The online version contains supplementary material available at 10.1186/s13063-021-05296-4.

## Background

Mild cognitive impairment (MCI) refers to cognitive impairment that falls between the cognitive changes of aging and early dementia [1, 2]. Patients with MCI have objective impairment in cognition that is not severe enough to require assistance with usual activities of daily living (ADL) [1, 2]. Patients with MCI have a 3–5-fold higher risk of developing any form of dementia in comparison to cognitively normal elderly persons [3–5]. Therefore, MCI has become a focus for trials attempting to either prevent or delay progression to AD [6].

Despite the importance of treating MCI, there is currently no established treatment method. There are no approved pharmacological treatments for MCI of any etiology, and only regular exercise training and cognitive training are recommended for these patients [2, 7]. MCI remains an active area of research, as numerous randomized controlled trials (RCTs) are being conducted to develop effective treatments [8].

Acupuncture treatment is widely used in Asia and can be considered an alternative treatment for MCI [9]. A meta-analysis involving 568 participants pooled from five RCTs researching acupuncture versus nimodipine treatment revealed that acupuncture was an effective treatment for amnestic MCI when used as an alternative or adjunctive treatment [10]. A meta-analysis of 5 RCTs with 257 patients investigating electroacupuncture (EA) versus western medications indicated that EA was effective in improving cognitive function in patients with MCI [11]. However, all clinical trials to date have used different acupuncture treatment methods. Acupuncture is a complex intervention of both, specific and non-specific factors associated with therapeutic benefit. Apart from needle insertion, issues such as needle sensation, psychological factors, acupoint specificity (AS), acupuncture manipulation, and needle duration (ND) also influence its therapeutic effects [12].

Although meta-analyses suggest that acupuncture may improve cognitive function in patients with MCI, the optimal acupuncture treatment method has not been established. Therefore, this RCT aimed to obtain basic data for developing an optimal acupuncture treatment for MCI, by investigating the factors contributing to cognitive improvement effects of acupuncture in these patients.

## Methods

This study design and reporting followed the standard protocol items of the Recommendations for Interventional Trials (SPIRIT) [13] and Consolidated Standards of Reporting Trials (CONSORT) statements [14]. The detailed methods of this study have been reported previously [15].

### Study design

This study was a prospective, outcome assessor-blinded, single-center (DongShin University Gwangju Korean Medicine Hospital, Republic of Korea) RCT with a 1:1:1:1 allocation ratio. A total of 32 participants who met the inclusion criteria were randomly allocated to basic acupuncture (BA), AS, ND, or EA groups (*n*=8/group). The patients received acupuncture treatment once/day, 3 days/week for 8 weeks.

The outcome measures were determined at baseline (week 0), 8 weeks after the first intervention (week 8; end of the intervention), and 12 weeks after completing the intervention (week 20). The study design is summarized in Table 1.

**Table 1:** Standard Protocol Items: Recommendations for Interventional Trials (SPIRIT) statement. The table shows the enrollment, interventions, and data collection protocols

	Study period										
	Enrolment	Allocation	Post-allocation								Close-out
Timepoint	Screening		Visit1–3	Visit4–6	Visit7–9	Visit10–12	Visit13–15	Visit16–18	Visit19–21	Visit22–24	Visit25
	Week		1	2	3	4	5	6	7	8	20
Enrolment											
Informed consent	X										
Sociodemographic profile	X										
Medical history	X										
Vital signs	X	X	X	X	X	X	X	X	X	X	X
Inclusion/exclusion criteria	X										
Allocation		X									
K-MMSE, MoCA-K	X										
Interventions											
Acupuncture treatment			X	X	X	X	X	X	X	X	
Assessments											
Change of medical history			X	X	X	X	X	X	X	X	X
Safety assessment			X	X	X	X	X	X	X	X	X
ADAS-K-cog			X							X	X
MoCA-K			X							X	X
CES-D			X							X	X
K-ADL, K-IADL			X							X	X
EQ-5D-5L			X							X	X

*K-MMSE*, Korean version of Mini-Mental State Examination; *MoCA-K*, Korean version of the Montreal Cognitive Assessment; *ADAS-K-cog*, Korean version of Alzheimer’s Disease Assessment Scale-cognitive subscale; *CES-D*, Center for Epidemiological Studies-Depression Scale; *K-ADL*, Korean Activities of Daily Living; *K-IADL*, Korean Instrumental Activities of Daily Living; *EQ-5D-5L*, European Quality of Life Five Dimension-Five Level Scale

### Ethical considerations

This study was conducted in accordance with the Declaration of Helsinki, and the study protocol (ver.1.0) was approved by the Institutional Review Board (IRB) of the DongShin University Gwangju Korean Medicine Hospital (DSGOH-051; approval date: September 17, 2018). This trial was registered at the Clinical Research Information Service (cris.nih.go.kr; registration number: KCT0003430; registration date: January 16, 2019) before the trial began. The purpose and potential risks of this study were fully explained to the participants. All participants provided written informed consent before participating in this study.

### Participant recruitment

Participants were recruited at the DongShin University Gwangju Korean Medicine Hospital in the Republic of Korea. The study recruitment was publicized via local newspapers, the Internet, and posters in communities and hospitals. Participants received an explanation regarding the study from the clinical research coordinator and were requested to voluntarily sign an informed consent form before participation. All potential participants were screened by the Korean versions of the Mini-Mental State Examination (K-MMSE) and the Korean versions of the Montreal Cognitive Assessment (MoCA-K) to confirm whether all inclusion criteria were met. The clinical research coordinator continuously monitored the medical conditions of the enrolled participants to maximize adherence to intervention protocols.

### Participation

The inclusion criteria were as follows: (1) age 55 to 85 years, (2) fulfillment of the Peterson diagnostic criteria for MCI [16] (subjective memory complaint, normal general cognitive function, objective memory impairment, not severe enough to interfere with ADL, and no dementia) with memory impairment for at least three months preceding enrollment, (3) K-MMSE scores of 20–23, (4) MoCA-K scale scores of 0–22, (5) Korean language fluency adequate for reliable completion of all study assessments, and (6) voluntary provision of informed consent.

The exclusion criteria were as follows: (1) diagnosis of dementia according to the Diagnostic and Statistical Manual of Mental Disorders-IV; (2) history of structural brain lesions that could cause cognitive impairment, such as traumatic brain injury, stroke, intracranial space-occupying lesions, or congenital mental retardation; (3) presence of cancer or serious cardiovascular, cerebrovascular, liver, or kidney diseases; (4) history of treatment for alcoholism, drug dependencies, or mental diseases, such as schizophrenia, serious anxiety, or depression in the 6 months preceding enrollment; (5) current treatment for MCI, such as medication, acupuncture, or cognitive training; (6) difficulties in assessment due to visual and hearing impairments; (7) presence of contraindications for acupuncture, such as blood clotting abnormalities (e.g., hemophilia), infection of the scalp, and presence of a pacemaker; and (8) concurrent participation in other clinical trials.

Participants were excluded from the trial under the following conditions: (1) less than 75% compliance with the protocol procedures (i.e., participating in fewer than 18 of the 24 scheduled treatment sessions), (2) reluctance to continue the trial, and (3) large error in the protocol or significant deviation in implementation.

### Randomization and blinding

Following the acquisition of written informed consent, the practitioners who would perform the intervention conducted a screening interview. Subsequently, the assessor performed baseline measurements for participants who met the inclusion criteria. The 32 enrolled participants were immediately assigned serial numbers generated using SPSS version 21 software (IBM Corp., Armonk, NY, USA) and randomly allocated to one of the four study groups (*n*=8/group). The serial number codes were inserted into opaque envelopes that were sealed and kept in a double-locked cabinet; these were opened by the principal investigator or practitioners who performed the intervention in the presence of the patient and a guardian.

We were able to adopt only a single outcome assessor-blinding approach, because sham treatment was not possible due to the characteristics of acupuncture application, which includes needle insertion and electric stimulation. During the study, the assessor was not in contact with any participant at any point other than the time of assessment, and data analysts with no conflicts of interest were involved in this study.

### Implementation

The clinical research coordinator generated the allocation sequence, enrolled the participants, and assigned them to intervention groups.

### Intervention

Participants received acupuncture treatment once/day, 3 days/week, for 8 weeks. The treatment was administered by Korean medicine doctors with 6 years of formal university training in Korean medicine and a license to administer treatment.

#### BA group

BA treatment was administered by acupuncture at Baihui (GV20), Sishencong (EX-HN1), Fengchi (GB20), and Shenting (GV24) [17, 18]. Only sterile, stainless steel, disposable acupuncture needles (size 0.25×30 mm; product no. A84010.02; Dong Bang Acupuncture, Inc., Boryeong, Republic of Korea) with guide tubes were used for treatment. With participants in the sitting position, needles were subgaleally inserted at an angle of 15–30° along the scalp. GB20 was punctured 17–30 mm in the direction of the tip of the nose. GV24, anterior EX-HN1, and GV20 were punctured in the forward direction, while the left, right, and posterior EX-HN1 were punctured in the direction of GV20. The depth of insertion was 9–24 mm, depending on the location of the needle [15, 19]. After insertion, the needles were left in position for 30 min; manual stimulation was not applied.

#### AS group

The AS acupuncture treatment method was the same as that of the BA group, except for additional acupuncture treatment at Taixi (KI3), where acupuncture needles were inserted bilaterally and vertically to a depth of 9–15 mm [19] for 30 min. KI3 is one of the most frequently used acupoints and, according to previous functional magnetic resonance imaging studies, can activate certain cognition-related regions [20–23].

#### ND group

The acupuncture treatment method for the ND group was the same as the BA group, except that the needles were retained for 20 min instead of 30 min.

#### EA group

The acupuncture treatment method for the EA group was the same as that of the BA group, except that it involved electrical stimulation of the acupoints using an EA stimulator (CELLMAC PLUS, STN-330; product no. A16010.04; Stratek, Co., Ltd., Anyang, Republic of Korea). GV24, GV20, EX-HN1 (left, right, anterior, and posterior), and GB20 (left and right) were administered EA using the following parameters: continuous waves, frequency 3–15 Hz, and intensity 2–4 mA [15, 17].

During the clinical trial period, all participants could continue their routine management regimens (including regular physical exercise) and existing medications. However, they were not permitted to engage in other treatments for ameliorating their MCI symptoms. All medical devices including the acupuncture needles and EA stimulator were inspected by the investigators, who recorded their findings in the management register.

### Outcome measurements

Scores for the Korean version of the Alzheimer’s Disease Assessment Scale-cognitive subscale (ADAS-K-cog), MoCA-K, Center for Epidemiological Studies-Depression Scale (CES-D), Korean Activities of Daily Living (K-ADL) scale, Korean Instrumental Activities of Daily Living (K-IADL) scale, and European Quality of Life Five Dimension Five Level Scale (EQ-5D-5L) were recorded before intervention (week 0), at the end of intervention (week 8), and at 12 weeks after intervention completion (week 20).

The ADAS-K-cog is a rating instrument commonly used to measure cognitive dysfunction in clinical trials and to detect, track, and stage AD [24]. In particular, it is known to be sensitive to the treatment responses of patients with MCI or early dementia [25].

The MoCA distinguishes patients with MCI from the normal population and has higher sensitivity in detecting cognitive decline than the MMSE [26].

The CES-D is a short self-rating 20-item scale designed to assess degrees of depressive symptoms and detect individuals at-risk for depression [27].

The K-ADL and K-IADL are used to assess physical function. The K-ADL and K-IADL were developed to assess basic activities of the elderly and estimate more complex activities necessary for independent daily life, respectively [28].

The EQ-5D-5L is a generic instrument used to measure health-related quality of life, comprising five dimensions [29].

### Sample size calculation

Due to the lack of adequate preliminary studies and limited research funds, study period, and recruitment opportunities, we adopted a pilot study design with eight participants in each of the four groups. As this was a pilot study, the sample size was insufficient for providing information on factors contributing to the cognitive improvement effects of acupuncture in patients with MCI. Thus, our study assessed the feasibility of arranging patients randomly in a trial of acupuncture for MCI; the results will be used to calculate sample size to provide a basis for subsequent research.

### Statistical analyses

The statistical analysis was revised in the study protocol, with IRB approval. We performed a full analysis (FA) set for the assessment of efficacy and a supplementary per-protocol (PP) analysis. Missing values were implemented by the last observation carried forward method. We compared the results of the FA set and PP analyses. Analyses were performed by blinded biostatisticians with SPSS version 20.0 software (SPSS Inc., Chicago, IL, USA) using two-sided significance tests with a 5% significance level. Continuous variables are presented as means and standard deviations (SD), and categorical variables are presented as count frequencies and percentages.

Data on baseline characteristics were collected and compared using an independent *t*-test, or a chi-squared (*χ*^2^) test. Differences between all outcome value changes in the four groups were compared using a Wilcoxon signed-rank test and repeated-measures analysis of variance (ANOVA) (Friedman tests). The ADAS-K-cog, MoCA-K, CES-D, K-ADL scale, K-IADL scale, and EQ-5D-5Lvalues were compared using repeated-measures ANOVA across two-to-three testing time points (weeks 0, 8, and 20). Differences in outcome value changes between the two groups (week 0 vs. week 8, week 0 vs. week 20, and week 8 vs. week 20) were compared using the Mann–Whitney *U* test (nonparametric test). Subanalysis of the study protocol was not conducted because the number of participants in the over 70 group was too small.

## Results

### Participants

**Fig. 1:**
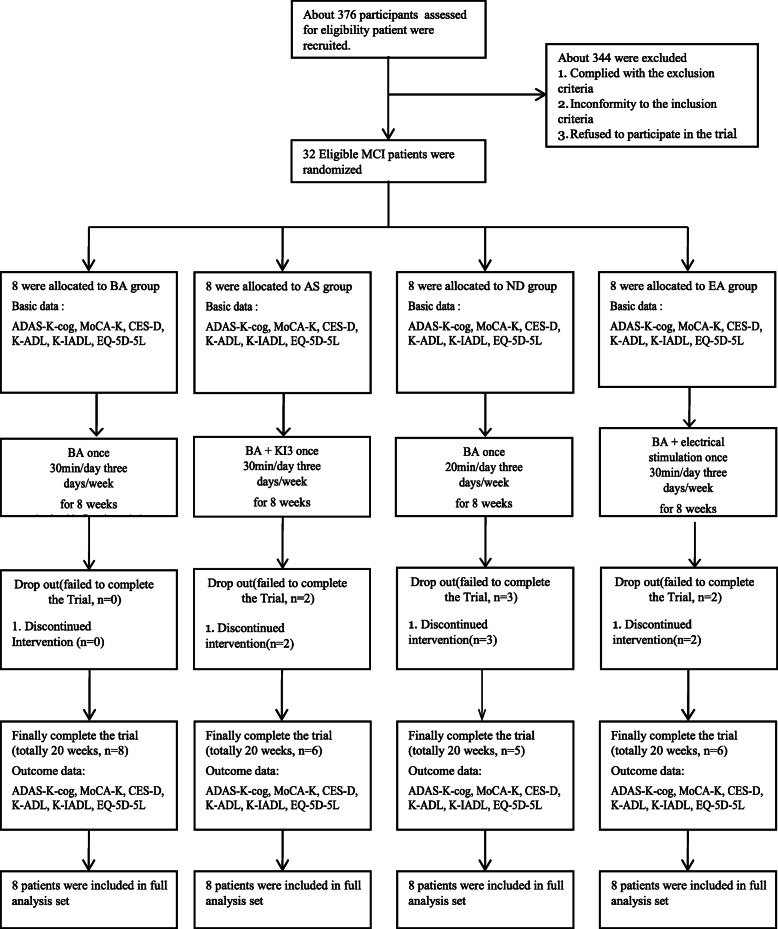
CONSORT 2010 flow diagram

We recruited participants between February 14, 2019, and December 20, 2019. During the study period, 376 patients were assessed for eligibility and 344 were excluded. Thirty-two patients were included in this study and were randomly assigned to the BA, AS, ND, or EA groups (*n*=8, each). Two participants in the AS and EA groups, and three in the ND group did not complete the treatment. The results of the FA set for the assessment of efficacy did not differ from those of the PP analysis. Thus, data for 32 patients with MCI were used in the final analysis (Fig. 1).

### Baseline characteristics

The baseline demographic characteristics and study variables for the 32 patients in the 4 groups are presented in Table 2. No significant differences were detected in the baseline demographic characteristics and study variables between the four groups (*p*>0.05; Table 2).

**Table 2:** Homogeneity tests for baseline demographic characteristics and study variables for 32 patients with mild cognitive impairment

Dependent variables	BA group (***n*** = 8)	AS group (***n*** = 8)	ND group (***n*** = 8)	EA group (***n*** = 8)	***F*** or *χ*^2^ (*p*)
	Mean (SD) or *n* (%)	Mean (SD) or *n* (%)	Mean (SD) or *n* (%)	Mean (SD) or *n* (%)	
Age (y)	67.25 (5.15)	65.00 (5.07)	70.75 (6.80)	64.63 (4.47)	51.87 (0.440)^a^
Sex (female)	7 (87.5%)	6 (75.0%)	7 (87.5%)	7 (87.5%)	0.71 (0.871)^a^
Education (years)	11.25 (5.95)	9.38 (2.50)	7.88 (1.36)	9.00 (2.27)	23.62 (0.312)^a^
MoCA-K	19.63 (1.51)	20.88 (1.36)	19.38 (2.13)	20.63 (2.07)	5.27 (0.153)^b^
ADAS-K-cog	12.13 (5.59)	7.00 (2.83)	10.13 (4.26)	9.13 (2.85)	5.11 (0.164)^b^
CES-D	15.63 (5.88)	15.13 (6.42)	15.25 (7.07)	15.50 (11.48)	0.30 (0.959)^b^
K-ADL	7.00 (0.00)	7.00 (0.00)	7.38 (0.74)	7.00 (0.00)	6.19 (0.103)^b^
K-IADL	10.75 (0.88)	11.00 (1.20)	11.88 (3.76)	10.25 (0.46)	2.32 (0.509)^b^
EQ-5D-5L	6.75 (1.28)	5.75 (0.87)	6.63 (1.60)	6.13 (0.84)	2.98 (0.395)^b^

^a^*x*^*2*^-test; ^b^*t*-test

### Efficacy of outcomes

After 8 weeks of intervention, we observed significant improvements in the BA group (changes in MoCA-K, ADAS-K-cog, and EQ-5D-5L scores), AS group (changes in MoCA-K and ADAS-K-cog scores), ND group (changes in MoCA-K score), and EA group (changes in ADAS-K-cog score) (Table 3).

**Table 3:** Changes in outcome measures (week 0 vs. week 8, week 0 vs. week 20) after treatment completion in the four groups

Groups	Dependent variables	Week 0 (M±SD)	Week 8 (M±SD)	Week 20 (M±SD)	Difference (W8–W0)	***Z*** (***p***)^a^	Difference (W20–W0)	***Z*** (***p***)^a^	***x***^**2**^ (***p***)^b^
BA group (*n*=8)	MoCA-K	19.63 ±1.51	25.75 ±1.75	25.75 ±2.82	6.13±1.81	−2.54 (0.011)	6.13±3.00	−2.53 (0.012)	12.80 (0.002)
	ADAS-K-cog	12.13 ±5.59	5.75 ±2.38	4.63±3.25	−6.38±5.71	−2.32 (0.021)	−7.50±5.37	−2.53 (0.012)	9.75 (0.008)
	CES-D	15.63 ±5.88	10.25 ±4.03	10.63 ±5.78	−5.38 ±5.37	−2.31 (0.021)	−5.00 ±7.67	−1.61 (0.108)	4.35 (0.114)
	K-ADL	7.00 ±0.00	7.00 ±0.00	7.00 ±0.00	0.00±0.00	0.00 (1.000)	0.00±0.00	0.00 (1.000)	0.00 (1.000)
	K-IADL	10.75±0.89	10.13 ±0.35	11.00±1.41	−0.63 ±1.06	−1.52 (0.129)	0.25±1.83	−0.34 (0.732)	3.39 (0.183)
	EQ-5D-5L	6.75 ±1.28	5.88 ±1.13	5.88 ±0.64	−0.88 ±0.64	−2.33 (0.020)	−0.88±1.13	−1.90 (0.058)	7.15 (0.028)
AS group (*n*=8)	MoCA-K	20.88 ±1.36	24.38 ±3.25	25.00 ±3.38	3.50±2.98	−2.21 (0.027)	4.13±3.36	−2.21 (0.027)	10.18 (0.006)
	ADAS-K-cog	7.00 ±2.83	4.50 ±1.93	3.38 ±1.77	−2.50±3.16	−1.90 (0.058)	−3.63±2.83	−2.21 (0.027)	7.64 (0.022)
	CES-D	15.13 ±6.42	9.50 ±6.91	13.38 ±9.38	−5.63 ±7.19	−2.00 (0.046)	−1.75 ±9.10	−0.73 (0.465)	4.36 (0.113)
	K-ADL	7.00 ±0.00	7.00 ±0.00	7.00 ±0.00	0.00.±0.00	0.00 (1.000)	0.00.±0.00	0.00 (1.000)	0.00 (1.00)
	K-IADL	11.00 ±1.20	10.63±1.06	10.75±1.39	−0.38±0.52	−1.73 (0.083)	−0.25±0.89	−0.81 (0.414)	2.36 (0.307)
	EQ-5D-5L	5.75 ±0.87	5.75 ±1.17	5.50 ±1.07	0.00±0.54	0.00 (1.000)	−0.25±0.71	−1.00 (0.317)	2.00 (0.368)
ND group (*n*=8)	MoCA-K	19.38 ±2.13	21.75 ±3.11	23.13 ±3.87	2.38±2.45	−2.02 (0.043)	3.75±3.33	−2.02 (0.043)	9.58(0.008)
	ADAS-K-cog	10.13 ±4.26	8.75 ±4.74	7.75 ±5.15	−1.38±3.85	−0.92 (0.357)	−2.38±2.62	−1.84 (0.066)	3.44 (0.179)
	CES-D	15.25 ±7.07	12.50 ±3.59	15.63 ±5.90	−2.75 ±7.15	−0.94 (0.345)	0.38±2.88	−0.27 (0.786)	2.80 (0.247)
	K-ADL	7.38 ±0.74	7.25 ±0.71	7.50 ±0.93	−0.13±0.35	−1.00 (0.317)	0.13±0.35	−1.00 (0.317)	2.00 (0.368)
	K-IADL	11.88±3.76	12.00 ±3.78	12.13±3.72	0.13 ±0.35	−1.00 (0.317)	0.25±0.46	−1.41 (0.157)	3.00 (0.223)
	EQ-5D-5L	6.63 ±1.60	6.13±1.36	5.88 ±1.46	−0.50 ±0.76	−1.63 (0.102)	−0.75±1.49	−1.30 (0.194)	2.38 (0.305)
EA group (*n*=8)	MoCA-K	20.63 ±2.07	22.50 ±2.73	23.25 ±3.28	1.88±2.10	−2.04 (0.041)	2.63±2.62	−2.00 (0.046)	5.48 (0.061)
	ADAS-K-cog	9.13 ±2.85	7.25 ±2.92	5.38 ±3.70	−1.88±2.10	−2.04 (0.041)	−3.75±2.38	−2.26 (0.024)	11.00 (0.004)
	CES-D	15.50 ±11.48	11.38 ±9.01	10.75 ±8.62	−4.13 ±6.40	−1.58 (0.114)	−4.75 ±4.30	−2.21 (0.027)	4.33 (0.142)
	K-ADL	7.00 ±0.00	7.00 ±0.00	7.00 ±0.00	0.00.±0.00	0.00 (1.000)	0.00.±0.00	0.00 (1.000)	0.00 (1.000)
	K-IADL	10.25±0.46	10.13 ±0.35	10.13±0.35	−0.13±0.64	−0.58 (0.564)	−0.13±0.64	−0.58 (0.564)	0.50 (0.779)
	EQ-5D-5L	6.13 ±0.84	5.63 ±0.74	5.50 ±0.76	−0.50±0.93	−1.41 (0.157)	−0.63±0.74	−1.89 (0.059)	5.20 (0.074)

^a^Wilcoxon signed-rank test; ^b^repeated-measures ANOVA (Friedman test)

Repeated-measures ANOVA revealed a significant interaction between time and group with respect to MoCA-K (*F*=2.56; *p*=0.029) and ADAS-K-cog scores (*F*=2.30; *p*=0.047) (Table 4).

**Table 4:** Results of repeated-measures ANOVA and the Scheffé post hoc test for the outcomes of treatment (*n*=32)

Dependent variables	Source of variation	SS	df	Mean square	***F***	***p***	Significant	Scheffé post hoc test ***F*** (***p***)		
								W8–W0	W20–W0	W20–W8
MoCA-K	Time	317.65	2	158.82	50.54	<0.001	S	5.11 (0.006)	1.79 (0.173)	0.69 (0.566)
	Group×time	48.35	6	8.01	2.56	0.029	S			
ADAS-K-cog	Time	313.90	2	156.95	26.46	<0.001	S	2.68 (0.066)	3.18 (0.039)	0.16 (0.920)
	Group×time	103.94	6	13.66	2.30	0.047	S			
CES-D	Time	325.90	2	162.95	6.66	0.003	S	0.32 (0.808)	1.26 (0.309)	0.60 (0.618)
	Group×time	103.94	6	17.32	0.71	0.708	NS			
K-ADL	Time	0.63	1.0	0.03	1.00	0.326	NS	1.00 (0.407)	1.00 (0.407)	1.00 (0.407)
	Group×time	0.19	3.0	0.62	1.00	0.407	NS			
K-IADL	Time	1.52	2	0.76	1.89	0.160	NS	1.73 (0.184)	0.45 (0.722)	1.74 (0.182)
	Group×time	2.65	6	0.44	1.10	0.375	NS			
EQ-5D-5L	Time	6.77	2	3.39	8.30	0.001	S	1.94 (0.146)	0.51 (0.676)	0.15 (0.931)
	Group×time	1.73	6	0.29	0.71	0.645	NS			

We conducted multiple comparisons of MoCA-K and ADAS-K-cog scores; significant interactions between time and group were observed in the ANOVA and Scheffé post hoc tests. MoCA-K scores were significantly increased in the BA group compared with the ND group (*p*=0.008, week 8–week 0) and EA groups (*p*=0.003, week 8–week 0; *p*=0.043, week 20–week 0). Additionally, ADAS-K-cog scores were significantly decreased in the BA group compared with the ND group (*p*=0.019, week 20–week 0) (Table 5).

**Table 5:** Multiple comparisons of MoCA-K and ADAS-K-cog scores among the four groups.

Groups	MoCA-K (***p***)^a^			ADAS-K-cog (***p***)^a^		
	W8–W0	W20–W0	W20–W8	W8–W0	W20–W0	W20–W8
BA vs AS	0.097	0.397	0.448	0.114	0.089	0.958
BA vs ND	0.008	0.314	0.128	0.063	0.019	0.874
BA vs EA	0.003	0.043	0.521	0.072	0.111	0.524
AS vs ND	0.422	0.707	0.498	0.418	0.384	0.784
AS vs EA	0.237	0.221	0.957	0.747	0.787	0.350
ND vs EA	0.745	0.335	0.514	0.322	0.248	0.305

^a^Mann–Whitney *U* test

### Safety evaluation

Adverse events that occurred in this study were recorded on a case report form after evaluating their relationships with the intervention. No adverse events related to the intervention occurred in this study.

## Discussion

Although acupuncture has long been used to treat cognitive dysfunction, the optimal acupuncture treatment method for cognitive impairment has not been established. To the best of our knowledge, this is the first RCT to investigate the factors including AS, ND, and electrical stimulation that contribute to the cognitive improvement effects of acupuncture in patients with MCI; this was performed by comparing the effects of the different acupuncture treatment methods. Our study is expected to provide the basis for developing an optimal acupuncture treatment method for MCI.

There were several main findings of our study. First, the BA group exhibited greater improvement in the MoCA-K score than the ND and EA groups at the end of the intervention. Second, the BA group exhibited greater improvement in the ADAS-K-cog scores than the ND group at 12 weeks after intervention completion. Third, we observed significant improvements in the BA group (changes in MoCA-K, ADAS-K-cog, and EQ-5D-5L scores), AS group (changes in MoCA-K and ADAS-K-cog scores), ND group (changes in MoCA-K scores), and EA group (changes in ADAS-K-cog scores). Thus, the four different acupuncture treatment methods resulted in significant cognitive improvement in patients with MCI, especially in the BA group, compared to the ND and EA groups.

EA sends weak electrical stimulation through acupuncture needles inserted in an acupoint; the synergistic effect of acupuncture and electrical stimulation can strengthen the overall stimulation [30]. Compared to manual acupuncture (MA), it has been demonstrated to produce a faster and better analgesic effect [31], greater improvement of limb motor function and ability of daily life activity in patients with acute ischemic stroke [32], greater effect on neuroblast plasticity in the dentate gyrus [33], more widespread signal increases in the human brain as measured by functional magnetic resonance imaging [34], and a greater improvement in frequency, loudness, and quality of life in patients with idiopathic tinnitus [35]. In contrast, no significant differences were reported in the effects of EA and MA in clinical trials for tinnitus treatment [36]. MA is more effective in reducing secondary menstrual symptoms in patients with primary dysmenorrhea compared to EA [37]. MA and EA may exert different therapeutic effects via different mechanisms related to the characteristics of diseases [38]. In our study, patients who received BA displayed a significant improvement in cognitive function compared to those who received EA. However, since this was a pilot study with a small sample size, it is difficult to conclude whether BA has a significant cognitive improvement effect compared to EA. Therefore, more rigorously designed comparative clinical studies are needed to obtain robust evidence for the cognitive improvement effects of BA and EA.

Needle duration refers to the amount of time the needle is held at an acupoint after needle manipulation, with the aim of awaiting Qi arrival, regulating Qi circulation, eliminating pathogenic factors, and reinforcing antipathogenic Qi and the effect of manipulation [12]. In our study, a 30-min ND resulted in a significant long-term cognitive improvement effect compared to the 20-min needle duration. Similarly, Bao et al. [39] previously reported that the longer the retention duration, the more significant the detected improvement in the myodynamia and hemorheology indices.

Acupoint specificity is an important basis for clarifying the functionality of acupoints. The selection and compatibility of acupoints are considered to directly affect its therapeutic effect [12]. In our study, the AS treatment method, with the additional KI3 acupoint, did not reveal a positive add-on cognitive improvement effect compared to the BA treatment method. This result is similar to that of a previous study by Zhang et al. [17] that reported that the addition of KI3, ST40, SP10, LR3, and ST36 to the scalp EA according to the traditional Chinese medicine syndromes did not significantly enhance the cognitive improvement effect compared to the conventional scalp EA treatment method.

Our study had some limitations. First, owing to the limited research funds, study period, and recruitment opportunities, the sample size was considerably small for investigating factors that contribute to cognitive improvement effects of acupuncture in patients with MCI. This may have led to a bias in the results. Second, we did not use various acupuncture doses to investigate the factors contributing to its cognitive improvement effects. Similar to medications, acupuncture has a dose-effect relationship [40]. A dose of acupuncture can be measured by the cumulative dose (frequency and total number of sessions), neurophysiological dose (number of needles, retention time, and mode of stimulation), or location of needles and treatment timing (before or during disease) [41]. There are various cumulative and neurophysiological doses and needle locations for treating MCI [10, 11]. However, the cumulative dose, neurophysiological dose, and needle locations used in this study were limited. Thus, further studies should be performed to investigate the effective dose of acupuncture. Finally, we adopted a single outcome assessor-blinding approach because double-blinding was impossible, given the characteristics of acupuncture application.

## Conclusions

Our results suggest that the four different acupuncture treatment methods have beneficial effects on cognitive function in patients with MCI. BA treatment resulted in greater cognitive improvement effects than ND and EA treatments and exerted the greatest cognitive improvement effect among the four groups. Electrical stimulation and needle duration may contribute to the cognitive improvement effects of acupuncture in patients with MCI. However, since our study is a pilot clinical trial with a small sample size, more rigorously designed clinical studies with a large sample size are needed to validate these results.

## Supplementary Information

**Additional file 1.** CONSORT 2010 Checklist.

**Additional file 2.** Revised Standards for Reporting Intervention in Clinical Trials of Acupuncture (STRICTA).

## Data Availability

The datasets used and/or analyzed during the current study are available from the corresponding author on reasonable request.

## Abbreviations

AD: Alzheimer’s disease
ADAS-K-cog: Korean version of Alzheimer’s Disease Assessment Scale-cognitive subscale
ANOVA: Analysis of variance
AS: Acupoint specificity
BA: Basic acupuncture
CES-D: Center for Epidemiological Studies-Depression Scale
EA: Electroacupuncture
EQ-5D-5L: European Quality of Life Five Dimension Five Level Scale
IRB: Institutional Review Board
K-ADL: Korean Activities of Daily Living scale
K-IADL: Korean Instrumental Activities of Daily Living scale
K-MMSE: Korean version of the Mini-Mental State Examination
MA: Manual acupuncture
MCI: Mild cognitive impairment
MoCA-K: Korean version of the Montreal Cognitive Assessment scale
ND: Needle duration
RCTs: Randomized controlled trials
