# Supplementary material for: Factors contributing to cognitive improvement effects of acupuncture in patients with mild cognitive impairment: a pilot randomized controlled trial
Source: Trials. 2021 May 12;22:341. doi: 10.1186/s13063-021-05296-4 (PMC8117619; doi:10.1186/s13063-021-05296-4)
Supplement: Supplementary file 2 — Additional file 2. Revised Standards for Reporting Intervention in Clinical Trials of Acupuncture (STRICTA). [file 13063_2021_5296_MOESM2_ESM.docx]

Revised Standards for Reporting Intervention in Clinical Trials of Acupuncture (STRICTA)

|  | **Item Criteria** | **Description** |
| --- | --- | --- |
| 1.Acupuncture rationale | 1a) Style of acupuncture | Korean Medicine Therapy |
|  | 1b) Reasoning for treatment provided – based on historical context, literature sources, and/or consensus methods, with references where appropriate | 1) Discussion among four doctors that practice Korean medicine (consensus)  2) Textbook of acupuncture and moxibustion medicine  3) Relevant articles [17-19]  Selection of treatment regions based on textbooks, related papers, and expert discussions |
|  | 1c) Extent to which treatment varied | Standardized treatment |
| 2. Details of needling | 2a) Number of needle insertions per subject per session (mean and range where relevant) | 8 or 10 |
|  | 2b) Names (or location if no standard name) of points used (uni-/bilateral) | Baihui(GV20), Sishencong(EX-HN1), Fengchi(GB20), and Shenting(GV24), Taixi(KI3) |
|  | 2c) Depth of insertion, based on a specified unit of measurement or on a particular tissue level | After the needles will be inserted into the acupoints subgaleally along the scalp at an angle of 15°-30°. GB20 will be punctured 17 –30mm in the direction toward nose tip. GV24, the anterior EX-HN1, and GV20 will be punctured forwards, and the left, right and posterior EX-HN1 toward GV20. The depth of insertion will be 9–24mm depending on the location of the needle. KI3 will be punctured bilaterally, vertically to a depth of 9-15mm[17,19] |
|  | 2d) Responses sought | No de qi or muscle twitching,– only sensation due to needle insertion |
|  | 2e) Needle stimulation | None or electrical stimulation |
|  | 2f) Needle retention time | 30-minute per session or 20-minute per session |
|  | 2g) Needle type | sterile, stainless, disposable acupuncture needles(size 0.25×30㎜; Dong Bang Acupuncture, Inc.,Boryeong, Republic of Korea; Product no: A 84010.02) |
| 3. Treatment regimen | 3a) Number of treatment sessions | 24 |
|  | 3b) Frequency and duration of treatment sessions | three times/week for 8weeks, 30-min per session or 20-min per session |
| 4. Other treatment components | 4a) Details of other interventions administered to the acupuncture group | none |
|  | 4b) Setting and context of treatment – including instructions to practitioners – as well as information and explanations given to patients | Practitioner-patient conversation about the context of the treatment, life habits, and daily life management |
| 5. Practitioner background | 5a) Description of participating acupuncturists | Korean medicine doctor with the following qualifications: 6 years of formal university training in Korean medicine, a license |
| 6. Control or comparator interventions | 6a) Rationale for the control or comparator in the context of the research question, with sources that justify the choice | Zhang H, Zhao L, Yang S, Chen Z, Li Y, Peng X, Yang Y, Zhu M. Clinical observation on effect of scalp acupuncture for mild cognitive impairment. J Tradit Chin Med. 2013;33(1):46-50 |
|  | 6b) Precise description of the control or comparator; details for items 1−3 above with the use of sham acupuncture or any other type of acupuncture-like control | This study will investigate the optimal acupuncture treatment method for the treatment of MCI through a comparison of the effects of different acupuncture treatment method according to acupoint specificity, needle duration, and electrical stimulation in terms of an improvement in cognitive function in patients with MCI. There is no control or comparator |
